# Evaluation of Zn, Cu, and Se Levels in the North American Autism Spectrum Disorder Population

**DOI:** 10.3389/fnmol.2021.665686

**Published:** 2021-04-29

**Authors:** Sunil Q. Mehta, Supriya Behl, Patrick L. Day, Adriana M. Delgado, Nicholas B. Larson, Lindsay R. Stromback, Andrea R. Huebner, Timothy R. DeGrado, Jessica M. Davis, Paul J. Jannetto, Flora Howie, Mukesh K. Pandey

**Affiliations:** ^1^Division of Developmental and Behavioral Pediatrics, Department of Pediatric and Adolescent Medicine, Mayo Clinic, Rochester, MN, United States; ^2^Department of Psychiatry and Psychology, Mayo Clinic, Rochester, MN, United States; ^3^Children’s Research Center, Department of Pediatric and Adolescent Medicine, Mayo Clinic, Rochester, MN, United States; ^4^Metals Laboratory, Department of Laboratory Medicine and Pathology, Mayo Clinic, Rochester, MN, United States; ^5^Department of Quantitative Health Sciences, Mayo Clinic, Rochester, MN, United States; ^6^Divisions of Nuclear Medicine and Research, Department of Radiology, Mayo Clinic, Rochester, MN, United States; ^7^Division of Community Pediatric and Adolescent Medicine, Department of Pediatric and Adolescent Medicine, Mayo Clinic, Rochester, MN, United States

**Keywords:** autism spectrum disorder, Zn, Cu, Se, biometals

## Abstract

Metal ion dyshomeostasis and disparate levels of biometals like zinc (Zn), copper (Cu), and selenium (Se) have been implicated as a potential causative factor for Autism Spectrum Disorder (ASD). In this study, we have enrolled 129 children (aged 2–4 years) in North America, of which 64 children had a diagnosis of ASD and 65 were controls. Hair, nail, and blood samples were collected and quantitatively analyzed for Zn, Cu and Se using inductively coupled plasma mass spectrometry (ICP-MS). Of the analyzed biometals, serum Se (116.83 ± 14.84 ng/mL) was found to be significantly lower in male ASD cases compared to male healthy controls (128.21 ± 9.11 ng/mL; *p* < 0.005). A similar trend was found for nail Se levels in ASD (1.01 ± 0.15 mcg/g) versus that of controls (1.11 ± 0.17 mcg/g) with a *p*-value of 0.0132 using a stratified Wilcoxon rank sum testing. The level of Se in ASD cohort was co-analyzed for psychometric correlation and found a negative correlation between total ADOS score and serum Se levels. However, we did not observe any significant difference in Zn, Cu, and Zn/Cu ratio in ASD cases versus controls in this cohort of North American children. Further studies are recommended to better understand the biology of the relationship between Se and ASD status.

## Introduction

Autism spectrum disorder (ASD) is a heterogeneous disorder characterized by both impaired social interaction, communication, and restricted, repetitive patterns in behavior (Lord et al., 2018). US prevalence has increased steadily since 2000: in 2016, the Centers for Disease Control and Prevention estimated 1 in 54 children have been diagnosed (Maenner et al., 2020). While the degree of impairment individually differs, the disorder is invariably life-affecting for families, and is considered a major public health issue (Newschaffer et al., 2007).

The etiology of ASD remains largely unknown; the condition is complex and caused by multiple factors. Initially, genetic factors were predominantly implicated in ASD incidence. Hundreds of genes related to ASD incidence have been identified in the literature, ranging in relatedness and pathogenicity. Genetic influence over ASD is heterogeneous and is seen in the form of rare chromosomal variants to common single nucleotide polymorphisms. Genes associated with ASD tend to peak in transcription during early fetal development, around the second trimester (Parikshak et al., 2013; Willsey et al., 2013). While this information may be clinically relevant (Vorstman et al., 2017), genetic variants only account for a portion of ASD susceptibility (Tick et al., 2016).

Consequently, the role of environmental factors in ASD incidence is widely researched. Most commonly, maternal factors such as maternal immune activation (Bilbo et al., 2018), preeclampsia, and gestational hypertension (Kim et al., 2019) have been noted as causative factors. Parental age at time of conception (Durkin et al., 2008) and maternal prenatal stress (Varcin et al., 2017) have also been implicated. The gut microbiome has been recently researched in the setting of ASD, and dietary changes have been suggested as potential treatment (Rosenfeld, 2015). However, much more research is needed to better understand the etiology of ASD (The Lancet and Neurology, 2017).

Metal micronutrients have been introduced as a potential causative factor in ASD. Biometals such as Zn, Cu, and Se are involved in neurodevelopment. Specifically, Zn is essential in neurodevelopment, and functions in postsynaptic plasticity and neurogenesis (Nam et al., 2017). A gene ontology analysis found a significant enrichment of Zn-binding domains in proteins involved in several aspects of all neurodevelopmental processes, including neuronal migration and neurotransmitter release (Behl et al., 2020). There is also an association between Zn and metallothioneins in ASD development. Treatment of ASD children with a Zn supplement can lead to higher cognitive-motor performance and serum metallothionein concentration (Meguid et al., 2019). Cu is a co-factor of several enzymes essential to the central nervous system (Lutsenko et al., 2010). Dyshomeostasis of Cu, particularly an excess of Cu, is a causative factor in several neurodegenerative diseases (Scheiber et al., 2014).

Se is another trace element for which homeostasis is essential to brain function. For instance, low Se levels are associated with faster decline of intellectual capacity in elderly individuals (Steinbrenner and Sies, 2013). Selenoproteins have protective antioxidant properties that prevent oxidative brain damage (Alim et al., 2019), an observation seen in children with ASD. Further, three selenoproteins (DIO1, 2, and 3) play a role in thyroid hormone regulation, which are involved in healthy brain development (Raymond et al., 2014). Se is also likely involved in various signaling processes of the developed brain, including the dopamine and GABA pathways (Solovyev, 2015). Interestingly, seven of the 25 selenoproteins peak in transcription by 4 years of age in a healthy child, and one of the genes GPX1 is directly associated with ASD susceptibility (Table 1). Taken together, Zn, Cu, and Se are evident in neurodevelopment and brain function, and may be implicated in ASD.

**TABLE 1:** 25 selenoproteins and characteristics.

Selenoprotein	Gene	Age at peak transcription	Autism-related gene?
**Glutathione peroxidases**			
Cytosolic GPx (cGPx, GPx-1)	*GPX1*	8 years	Yes
Gastrointestinal GPx (GI-GPx, GPx-2)	*GPX2*	Unknown	
Plasma GPx (pGPx, GPx-3)	*GPX3*	37 pcw	
Phospholipid hydroperoxide GPx (PHGPx, GPx-4)	*GPX4*	4 years	
Sperm nuclei GPx (snGPx)	*GPX4*		
GPx-6	*GPX6*	Unknown	
**Thioredoxin reductases**			
Thioredoxin reductase 1 (TrxR1)	*TXNRD1*	Unknown	
Thioredoxin reductase 2 (TrxR2, SelZf1, SelZf2)	*TXNRD2*	Unknown	
Thioredoxin reductase 3 (TrxR3, TGR)	*TXNRD3*	Unknown	
**Iodothyronine deiodinases**			
Type 1 deiodinase (DIO1, IOD1, D1)	*DIO1*	Unknown	
Type 2 deiodinase (DIO2, IOD2, D2)	*DIO2*	4 months	
Type 3 deiodinase (DIO3, IOD3, D3)	*DIO3*	12 pcw	
Selenophosphate synthetase (SPS2)	*SEPHS2*	Unknown	
Selenoprotein S (SelS, VIMP)	*SEPS1*	Unknown	
Selenoprotein P (SEPP1, SelP)	*SEPP1*	8, 21 years	
Selenoprotein 15 kDa (Sel15)	SEP15	4 years	
Selenoprotein N (SelN)	*SEPN1*	Unknown	
Selenoprotein X (SelX or SelR)	*SEPX1*	8 years	
Selenoprotein W (SelW)	*SEPW1*	4–8 years	
Selenoprotein T (SelT)	SEPT1	Unknown	
Selenoprotein H	SELH	4 years	
Selenoprotein I	SELI	Unknown	
Selenoprotein K	SELK	3–4 years	
Selenoprotein M	SELM	8 years	
Selenoprotein O	SELO	Unknown	
Selenoprotein V	SELV	Unknown	

*The age at which the transcript peaks in transcription levels are indicated, if evident through BrainSpan Atlas of the Developing Human Brain© 2010 (Allen Institute for Brain Science. Available through: https://www.brainspan.org/). Autism-related genes were identified through the SFARI Gene Database 2020 (Available through: https://gene.sfari.org/).*

Previous studies have compared levels of Zn, Cu, and Se in children with and without ASD (Faber et al., 2009; Lakshmi Priya and Geetha, 2011; Russo and Devito, 2011), which identified metal dyshomeostasis in children with autism. However, studies often only use one matrix (serum, hair, or nail samples), leading to incomparable results. Metal micronutrient level difference in the ASD setting can also be attributed to geography and social determinants of health, as well as age variability between studies. Many studies report relatively small sample sizes, leading to conflicting data. Lastly, results are rarely stratified by sex in previous studies, although there is a need for sex stratification in metal micronutrient analysis in ASD (Dickerson et al., 2017; Behl et al., 2020). Although the pathophysiology of ASD is heterogenous, differences in neural development are almost always apparent during the first years of life. A more comprehensive examination into metal micronutrient levels in young children with ASD is therefore needed to better understand ASD etiology and strategies for treatment. In the present study, the sex-specific levels of Zn, Cu, and Se are evaluated in a large cohort of young children in the United States diagnosed with ASD along with healthy controls.

## Materials and Methods

### Patient Recruitment

Children aged 24–48 months newly diagnosed with ASD were recruited into the study from the Dana Neurodevelopmental Disorders program of Mayo Clinic (Rochester, Minnesota), along with age-matched controls seen in an outpatient community pediatrics clinic. All case and control samples were collected through informed consent approved by the local institutional review board (Mayo Clinic Institutional Review Board). Methods were carried out in accordance with relevant guidelines and regulations.

### Sample Collection

Serum samples were collected using BD royal blue clot activator vacutainer tubes (Franklin Lakes, NJ), allowed to clot, centrifuged, poured over into metal-free specimen vials, and shipped at 4°C to the metals laboratory for analysis. Hair from the back of the head and all 10 fingernails were collected and put into metal-free specimen containers and also sent to the metals laboratory at ambient temperature.

### Analytical Method and Chemicals

#### Inductively Coupled Plasma Mass Spectrometry (ICP-MS)

All elemental analysis was performed in an ISO class 7 cleanroom laboratory to reduce the risk of potential metal contamination. All inductively coupled plasma mass spectrometry experiments were performed using PerkinElmer Elan or PerkinElmer NexION 350D ICP-MS spectrometers (Waltham MA) equipped with Elemental Scientific Inc. (ESI) SC2-DX autosamplers (Omaha NE). The mass spectrometers were equipped with ESI microflow PFA-ST nebulizers and quartz cyclonic spray chambers including a baffle. For the analysis of Se, Cu, and Zn in hair and nail samples, grade 5 helium, purchased from Praxair (Danbury, CT) was used as an inert gas in kinetic energy discrimination mode (KED). For the analysis of Se, Cu, and Zn in serum samples, grade 5 ammonia, purchased from Praxair was used as a reactant gas in dynamic reaction mode (DRC).

#### Hair and Nail Digestion

Within the laboratory, human hair and nail samples were first thoroughly washed with 0.1% Triton-X-100 (Dow Chemical Midland, MI), and then with reagent grade water in order to remove external contaminants. The samples were then allowed to completely dry in a 90°C oven. Dried hair and nail samples were then weighed and digested using 0.5 mL of trace metal grade 65% nitric acid (Fisher Scientific, Waltham, MA) on a dry bath incubator set to 80°C. The target sample weight of 0.05–0.10 g of hair or nails was used for sample digestion. After complete digestion, 0.5 mL of a 1% HNO_3_ solution was added to make a final volume of 1.0 mL. Finally, prior to analysis, an internal standard solution containing gallium was added to the sample.

#### Isotopes

For calibration and analysis of Se, Cu and Zn in serum the following isotopes were quantitated: ^82^Se, ^65^Cu, and ^64^Zn. ^71^Ga was used as an internal standard for this assay. For calibration and analysis of Se, Cu and Zn in hair and nail samples, the following isotopes were quantitated: ^78^Se, ^65^Cu, ^64^Zn. For the measurement of Se in hair and nail samples, ^69^Ga was used as an internal standard. For the measurement of Cu and Zn in hair and nail samples, ^71^Ga was used as an internal standard.

#### Reagents

Salt based calibration standards containing Se, Cu, and Zn were purchased from Inorganic Ventures (Christiansburg VA). Trace metals grade 65% nitric acid and trace metal grade 38% hydrochloric acid were purchased from Thermo Fisher Scientific and used in the creation of reagents. ACS grade tert-butanol was purchased from ACROS Organics (Fairlawn, NJ) and was used in the creation of reagents. Reagent quality water was purified using a Barnstead Nanopure Diamond water purification system purchased from Thermo Fisher Scientific.

#### Reference Material and Quality Control Samples

For the analysis of hair and nail samples, the National Institute for Standards and Technology Material 2976 Mussel Tissue Powder (Gaithersburg MD) and NCS ZC 81002b Human Hair (Beijing China) were analyzed with each calibration and with each sample run to monitor the accuracy of the methods. Reference materials were stored as recommended by the product inserts.

For the analysis of serum, Utak Trace Elements Serum Toxicology controls (Valencia, CA) were analyzed with each calibration and subsequent sample runs to monitor accuracy of the method. Utak control materials were stored as recommended by the product inserts.

#### Analysis

For the analysis of Se in serum, hair and nail, six calibration standards were used to establish an analytic measurement range of 0–500 ng/mL, whereas, for Cu and Zn analysis in serum, six calibration standards used were in the range of 0–5,000 ng/mL. Reagent grade water was analyzed as reagent blanks during the calibration process to establish instrument and reagent cleanliness.

For the analysis of Cu in hair and nails, seven calibration standards were used to establish an analytical measurement range of 0–1,000 ng/mL. For the analysis of Zn in hair and nails, seven calibration standards were used to establish an analytic measurement range of 0–10,000 ng/mL. Reagents blanks consisting of dilute nitric acid were analyzed during the calibration process to establish instrument and reagent cleanliness.

For all calibration curves, minimum linear regression acceptability was set at a regression coefficient of ≥ 0.998. All samples were diluted 1 × 25 using an assay specific diluent and vortexed before placement on the autosampler and subsequent analysis.

All assays were developed, and their performance characteristics determined in a manner consistent to meet Clinical Laboratory Improvement Amendments (CLIA) requirements. The laboratory developed tests were fully validated and correlated well with equivalent outside reference laboratory tests.

### Statistical Analysis

Study data were recorded and managed using the Research Electronic Data Capture system (Harris et al., 2009). Quantitative variables were summarized by median ± standard deviation (SD), while categorical variables were summarized as counts and percentages. For comparisons of metal ion measurements by case-status, a stratified Wilcoxon rank sum test (stratified by sex) was performed, as well as sex-specific analyses using the standard Wilcoxon test. All hypothesis testing was conducted under a two-sided alternative. Correlations of metal levels with psychometric testing results and body mass index were evaluated using Kendall’s Tau rank correlations with a two-sided test of significance. For all hypothesis testing, a nominal *p* < 0.05 was considered statistically significant. No adjustments were made for multiple testing, given the correlated nature of the biomarkers. All analyses were performed using the R v3.6.2 statistical software package (R Core Team, Vienna, Austria) (R Core Team, 2017).

## Results

### Patients and Samples

A total of 129 children were enrolled, 64 had a diagnosis of Autism Spectrum Disorder and 65 were controls (Table 2). There were a slightly higher proportion of females in the control group; though this was difference was not statistically significant. Ages in both groups ranged from 24 to 47 months (median = 36 months), with controls being younger (*p* = 0.04). Height and weight differed between groups, likely due to this difference in age. The majority of patients self-identified as White and not of Hispanic or Latinx ethnicity. The median height and weight across groups were 95.4 cm and 15.1 Kg, respectively, with a median body mass index (BMI) of 16.68 kg/m^2^ at around the time of sample collection.

**TABLE 2:** Characteristics of case and control study subjects.

	ASD (*N* = 64)	Control (*N* = 65)	Total (*N* = 129)	*p*-value
**Age (months)**				0.022
Median (SD)	38.5 (7.73)	36 (6.62)	36 (7.29)	
**Sex**				<0.001
Female	10(15.6%)	30(46.2%)	40(31.0%)	
Male	54(84.4%)	35(53.8%)	89(69.0%)	
**Race**				0.153
Asian	3(4.7%)	1(1.5%)	4(3.1%)	
Black or African American	4(6.2%)	0(0.0%)	4(3.1%)	
More than one race	0(0.0%)	1(1.5%)	1(0.8%)	
Unknown/Not reported	10(15.6%)	14(21.5%)	24(18.6%)	
White	47(73.4%)	49(75.4%)	96(74.4%)	
**Ethnicity**				0.103
Hispanic or Latino	8(12.5%)	2(3.1%)	10(7.8%)	
NOT Hispanic or Latino	45(70.3%)	46(71.9%)	91(71.1%)	
Unknown/Not reported	11(17.2%)	16(25.0%)	27(21.1%)	
**Height (cm)**				<0.001
Median (SD)	96.75 (6.13)	94 (5.71)	95.4 (6.24)	
**Weight (kg)**				<0.001
Median (SD)	15.7 (3.01)	14.1 (1.93)	14.6 (2.70)	
**BMI (kg/m^2^)**				0.129
Median (SD)	16.69 (2.01)	16.35 (1.30)	16.5 (1.72)	

*Quantitative variables are presented as median ± SD, whereas categorical variables are presented as counts (percentages).*

The ASD cohort had occasional co-diagnoses, such as attention-deficit/hyperactivity disorder (6%), intellectual disability (2%), speech delay (69%), disruptive behavior disorder (2%), and global developmental delay (6%).

### Serum Analysis of Zn, Cu, and Se

**FIGURE 1:**
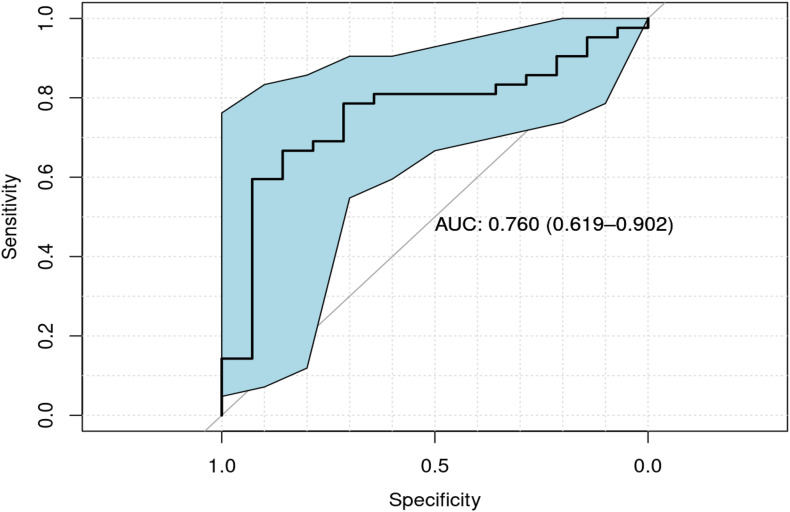
ROC curve of serum Se values for cases vs. controls among males, along with confidence interval regions for sensitivity over a grid of specificities (indicated in shaded blue region). An estimate of the AUC and its 95% confidence interval (in parentheses) is also provided.

Of the 129 children enrolled, 74 provided a blood sample (52 children with ASD and 22 controls). Overall and sex-stratified comparisons were made between cases and controls (Table 3). Of these comparisons, median serum Se levels were lower in cases than controls, with significant difference among males (*p* = 0.004). A Receiver Operating Characteristic (ROC) curve analysis Under Curve (AUC) of 0.760 (95% CI = [0.619–0.902]; Figure 1). Among controls, there was a significant difference in serum Se levels by sex (Table 3; 109.41 ng/mL in females vs. 128.21 ng/mL in males; *p* < 0.001).

**TABLE 3:** Median Cu, Zn, and Se levels for cases and controls, stratified by sex.

	ASD (*N* = 52)	Control (*N* = 22)	*p*-value
**Mean Cu (SD)**	1.37 (0.26)	1.32 (0.19)	0.75642
Female	1.29 (0.20)	1.23 (0.08)	0.85867
Male	1.39 (0.27)	1.38 (0.21)	0.99245
**Mean Zn (SD)**	0.75 (0.16)	0.81 (0.30)	0.57792
Female	0.74 (0.15)	0.69 (0.08)	0.4239
Male	0.75 (0.16)	0.88 (0.35)	0.23092
**Mean Se (SD)**	116.67 (14.75)	121.37 (13.69)	0.07166
Female	115.96 (15.16)	109.41 (12.29)	0.32838
Male	116.83 (14.84)	128.21 (9.11)	**0.00379**

*Median Cu, Zn, and Se levels for cases and controls, stratified by sex. Serum zinc and copper levels are presented in units mcg/mL. Serum selenium levels are presented in units ng/mL. Bolded p-value denotes significance.*

There were no significant differences in median serum Zn or Cu levels. The Zn/Cu ratio was similarly evaluated with no significant differences between cases and controls.

### Hair and Nail Analysis of Se

A total of 66 participants provided hair samples (26 ASD, 40 controls), and 62 provided nail samples (23 ASD, 39 controls). Only one female with ASD provided hair and nail samples, therefore comparisons were made only between males (Table 4). Nail Se levels were significantly lower in ASD cases than in controls in males (*p* = 0.013). In hair samples, median Se levels were similarly higher among cases than controls, albeit no significant difference was observed (*p* = 0.299).

**TABLE 4:** Median Se levels between cases and controls, stratified by sex.

	ASD	Control	*p*-value
**Hair Se (SD)**	0.58 (0.13)	0.66 (0.20)	
Female	0.59(*NA*)	0.68 (0.26)	–
Male	0.58 (0.14)	0.63 (0.11)	0.2992
**Nail Se (SD)**	1.00 (0.14)	1.11 (0.16)	
Female	0.96(*NA*)	1.11 (0.15)	–
Male	1.01 (0.15)	1.11 (0.17)	**0.0132**

*Median Se levels between cases and controls, stratified by sex. Hair and nail levels are presented in units mcg/g. Bolded p-value denotes significance. 66 participants provided hair samples (26 ASD, 40 controls), and 62 provided nail samples (23 ASD, 39 controls). Only one ASD female provided hair/nail samples in this study, therefore female-specific comparisons were not conducted.*

### Psychometric Testing Correlations

**FIGURE 2:**
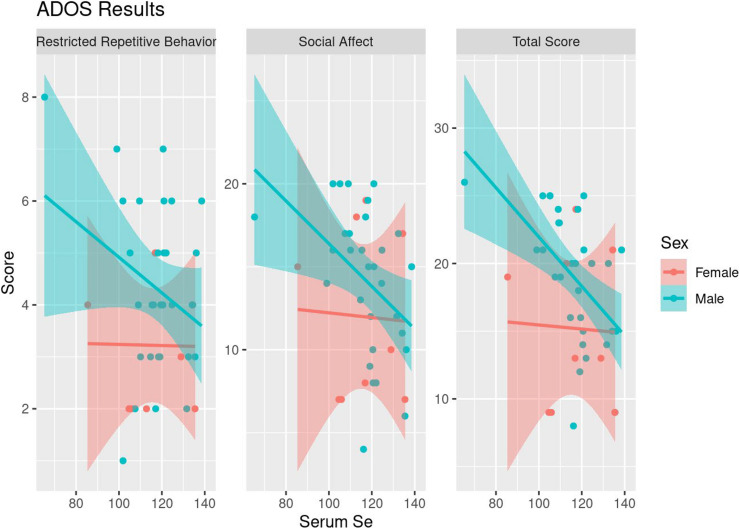
Scatterplots of ADOS Results and serum Se levels along with fitted linear trends, stratified by sex.

Among the 52 children with ASD who provided serum samples, correlation analyses were conducted between serum levels of Zn, Cu, and Se with psychometric testing scores (Vineland-3, ADOS-2, and the Preschool Language Scale). Significant correlations were not identified between psychometric testing and Zn or Cu levels. However, a significant negative correlation was seen between ADOS total score and serum Se levels (τ = −0.260, *p* = 0.02), particularly in males (τ = −0.404, *p* = 0.002; Table 5 and Figure 2). This was largely driven by a significant negative correlation between the Restrictive Repetitive Behavior portion of the ADOS test and serum Se levels in males (τ = −0.350, *p* = 0.008).

**TABLE 5:** Tau rank correlations between various psychometric test scores and serum Se levels, in all patients and males only.

Test	Value	Sample size (All)	τ (All)	*P* (All)	Sample size (Males)	τ (Males)	*P* (Males)
Vineland	Communication	30	0.135	0.3	25	0.149	0.303
Vineland	Daily living skills	31	0.162	0.207	26	0.072	0.611
Vineland	Socialization	28	0.145	0.285	23	0.116	0.443
Vineland	Motor skills	28	0.032	0.812	24	−0.011	0.941
ADOS	Restricted repetitive behavior	39	−0.216	0.058	30	−0.35	0.008
ADOS	Social affect	39	0.016	0.892	30	−0.017	0.899
ADOS	Total score	41	−0.26	0.02	32	−0.404	0.002
Preschool language scale	Auditory comprehension	30	0.118	0.369	22	0.102	0.514
Preschool language scale	Expressive communication	30	0.111	0.399	22	0.185	0.242

### Analysis of Se and BMI

**FIGURE 3:**
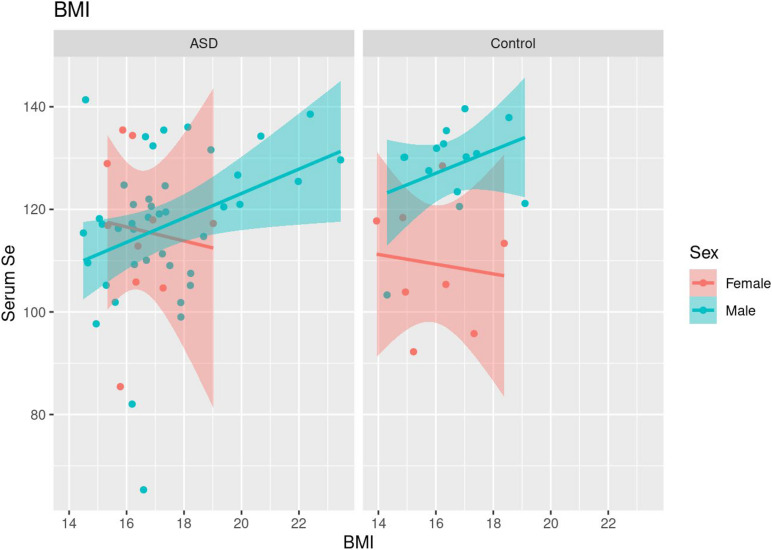
Scatterplots of BMI and serum Se levels along with fitted linear trends, stratified by sex.

In both cases and controls, a positive correlation was seen between body mass index (BMI) and serum Se levels in males (τ = 0.24, *p* = 0.025; Table 6 and Figure 3). Females did not demonstrate a significant correlation between BMI and serum Se levels in sex-specific analyses.

**TABLE 6:** Kendall’s Tau rank correlation between serum Se levels and BMI, overall and stratified by sex and case-status.

Sex	Case status	τ	*P*-value
All	All	0.134	0.091
Male	All	0.166	0.07
Female	All	−0.098	0.601
All	ASD	0.165	0.084
Male	ASD	0.24	0.025
Female	ASD	−0.156	0.601
All	Control	0.221	0.16
Male	Control	0.231	0.279
Female	Control	−0.143	0.72

## Discussion

The findings of this study indicate a significant association between lower Se levels and the presence of autism spectrum disorder. Se, along with their associated selenoproteins, are critical to the function of many biological processes such as formation of thyroid hormones, DNA synthesis, and fertility (Mehdi et al., 2013). Se is involved in immune function through activated T cells. Studies show that Se supplementation leads to faster and more effective immune response (Rayman, 2012). It remains evident that Se is also integral to brain function. Selenoprotein P (SEPP1) is responsible for delivery of Se to the brain and plays a neuroprotective role against oxidative stress. SEPP1-deficient mice exhibits clear neurological dysfunction. Lack of dietary Se in these mice led to brain injury and subsequent death (Hill et al., 2004).

A majority of selenoproteins are involved in response to oxidative stress (Raymond et al., 2014), and antioxidant response is critical to neurodevelopment. Increased oxidative stress has been frequently reported in children with ASD (Bjorklund et al., 2020), through reactive oxygen species (Yui et al., 2016), increased lipid peroxidation (Ming et al., 2005), and reduced levels of antioxidants (Sogut et al., 2003). Interestingly, one antioxidant consistently reduced in ASD children is glutathione peroxidase-1 (GPx1), a selenoprotein and known ASD-related gene (Ming et al., 2010). More recently, Se has been shown to block a programmed cell death pathway activated in response to oxidative stress called ferroptosis (Alim et al., 2019). Although ferroptosis has primarily been studied in adults with stroke, TBI, and Parkinson’s disease, it is possible this mechanism is involved in neural development as well. Thus, it is possible that insufficient Se increases neuronal sensitivity to oxidative stress in children with ASD.

Of note, this study found significantly lower levels of Se in serum and nail samples, but not in hair samples. While blood samples consistently demonstrate lower Se levels in ASD cases (Jory and McGinnis, 2008; Skalny et al., 2017a), hair samples vary in Se levels when compared in ASD and controls (Raymond et al., 2014). Blood Se levels are more indicative of tissue selenoenzyme activities and are considered more reliable than levels taken from other biospecimens (Raymond et al., 2014; Pozebon et al., 2017). Differences in variation suggest hair and nail samples are more susceptible to environmental factors such as consumer product use. For example, many anti-dandruff shampoos include high Se levels. Of note, this study recorded participants’ usage of consumer products high in Zn, Cu, or Se. Participants using such products were eliminated from hair and nail sample analysis.

An additional finding of this study is the sex-specific effects of Se levels, in both cases and controls. Credible arguments have been made to stratify metal micronutrient data by sex, as there are many key sex differences that could confound results if sexes are combined (Dickerson et al., 2017; Behl et al., 2020). One study found that stratifying metal micronutrient levels by both sex and age identified key findings. In particular, Se levels were significantly lower in young children (ages 2–5 years) with ASD when age was stratified, consistent with the results of this study. Further, stratifying results by sex identified several novel associations (Skalny et al., 2017b). While our study found key differences among males, very few female ASD patients were enrolled, and therefore more work should be done to evaluate female-specific Se levels between ASD cases and controls. Further, our study identified significant differences among controls between males and females, and should be examined further in the context of reference ranges.

This study also identified a correlation between BMI and Se levels within males. This is consistent with previous findings in older children; a negative correlation has been seen between Se levels and overweight or obese patients, particularly those with an ASD diagnosis (Blazewicz et al., 2020). The underlying mechanism behind this is not clear, though suggestions have been made that food selectivity is higher in children with ASD, therefore Se deficiency may be the result of differences in food consumption (No Author List, 2020). It is also possible that Se deficiency may contribute to obesity through altered thyroid function, although this hypothesis has not been examined directly to our knowledge.

Contrary to prior literature, we did not identify significance differences of Zn and Cu levels between ASD cases and controls. Both Zn deficiency and increased Cu levels have been implicated to affect the presence and severity of ASD. However, as previously noted, geographical differences are seen in Zn and Cu levels. Further, significant differences have not been seen in Zn or Cu between ASD cases and controls in the North American population (Behl et al., 2020). The cohort in this study may not have had sufficient power to identify similar effects. Much variability was seen among metal ion levels, and a larger cohort of both ASD cases and controls should be evaluated to reproduce these findings.

This study includes a large cohort (129 children) to evaluate the role of metal ions like Zn, Cu and Se in ASD. Further, our cohort was comprised of a more narrow age range of children that correspond to the symptom onset of ASD, in contrast to broader cross-sections studied previously. It would be important for future studies to examine biometal levels early in development in order to establish a role in ASD etiology. However, some limitations to this study exist. First, the study is cross-sectional, and therefore causality is difficult to establish. The disparities identified in this protocol could be due to several factors, including restricted diet and/or gastrointestinal implications for ASD. Second, while this study was one of the first to identify sex-dependent differences in metal ion levels, there was a limited number of females enrolled, leading to inconclusive results in females. Lastly, the cohort in this study may not be representative of the racial and ethnic diversity within North America, as majority of participants identified as White.

This study identified a prominent deficiency of Se in boys aged 2–4 years with ASD. The results of this study open up opportunities for future research in Se and autism spectrum disorder. The biology of Se and its association with neurodevelopment is apparent, but more research should be done to further solidify the mechanism of action.

## Conclusion

We identified significantly lower levels of serum and nail Se in male ASD cases compared to male controls. We also found a negative correlation of Se levels in male ASD group with total ADOS score. Although total ADOS score is not a validated measure of symptom severity, we did see a strong association between lower serum Se levels and more restricted and repetitive behaviors in males.

The role of Se in human physiology is not fully understood. However, there are a significant number of findings that show Se involved in various biological functions such as DNA synthesis and thyroid hormone synthesis, and also plays an important role in immune response and neuroprotection against oxidative stress. Animal studies showing lack of dietary Se resulting in brain injuries further supports the notion that Se plays a critical role in protecting the brain against oxidative stress. Our findings clearly warrant further studies to better understand the physiological role of Se and other biometals in ASD through sex-based stratified analysis.

## Data Availability Statement

The original contributions presented in the study are included in the article/supplementary material, further inquiries can be directed to the corresponding author/s.

## Ethics Statement

The studies involving human participants were reviewed and approved by all case and control samples were collected through informed consent approved by the local institutional review board (Mayo Clinic Institutional Review Board). Written informed consent to participate in this study was provided by the participants’ legal guardian.

## Author Contributions

All authors have contributed in the manuscript either in patient recruitments, data generation, data analysis, method development or manuscript writing, and editing.

## Conflict of Interest

The authors declare that the research was conducted in the absence of any commercial or financial relationships that could be construed as a potential conflict of interest.

## References

[B1] Alim I. Caulfield J. T. Chen Y. Swarup V. Geschwind D. H. Ivanova E. (2019). Selenium drives a transcriptional adaptive program to block ferroptosis and treat stroke. *Cell* 177 1262–1279 e25. 31056284 10.1016/j.cell.2019.03.032

[B2] Behl S. Mehta S. Pandey M. K. (2020). Abnormal levels of metal micronutrients and autism spectrum disorder: a perspective review. *Front. Mol. Neurosci.* 13:586209. 10.3389/fnmol.2020.586209 PMC7759187 33362464

[B3] Bilbo S. D. Block C. L. Bolton J. L. Hanamsagar R. Tran P. K. (2018). Beyond infection - Maternal immune activation by environmental factors, microglial development, and relevance for autism spectrum disorders. *Exp. Neurol.* 299(Pt A), 241–251. 10.1016/j.expneurol.2017.07.002 28698032 PMC5723548

[B4] Bjorklund G. Meguid N. A. El-Bana M. A. Tinkov A. A. Saad K. Dadar M. (2020). Oxidative stress in autism spectrum disorder. *Mol. Neurobiol.* 57 2314–2332. 32026227 10.1007/s12035-019-01742-2

[B5] Blazewicz A. Szymanska I. Dolliver W. Suchocki P. Turlo J. Makarewicz A. (2020). Are obese patients with autism spectrum disorder more likely to be selenium deficient? research findings on pre- and post-pubertal children. *Nutrients* 12:3581. 10.3390/nu12113581 33266486 PMC7700552

[B6] Dickerson A. S. Rotem R. S. Christian M. A. Nguyen V. T. Specht A. J. (2017). Potential sex differences relative to autism spectrum disorder and metals. *Curr Environ. Health Rep.* 4 405–414. 10.1007/s40572-017-0164-x 28988324 PMC6508872

[B7] Durkin M. S. Maenner M. J. Newschaffer C. J. Lee L. C. Cunniff C. M. Daniels J. L. (2008). Advanced parental age and the risk of autism spectrum disorder. *Am. J. Epidemiol.* 168 1268–1276. 18945690 10.1093/aje/kwn250 PMC2638544

[B8] Faber S. Zinn G. M. Kern J. C. Kingston H. M. (2009). The plasma zinc/serum copper ratio as a biomarker in children with autism spectrum disorders. *Biomarkers* 14 171–180. 10.1080/13547500902783747 19280374

[B9] Harris P. A. Taylor R. Thielke R. Payne J. Gonzalez N. Conde J. G. (2009). Research electronic data capture (REDCap)–a metadata-driven methodology and workflow process for providing translational research informatics support. *J. Biomed. Inform.* 42 377–381. 10.1016/j.jbi.2008.08.010 18929686 PMC2700030

[B10] Hill K. E. Zhou J. McMahan W. J. Motley A. K. Burk R. F. (2004). Neurological dysfunction occurs in mice with targeted deletion of the selenoprotein P gene. *J. Nutr.* 134 157–161. 10.1093/jn/134.1.157 14704310

[B11] Jory J. McGinnis W. R. (2008). Red-cell trace minerals in children with Autism. *Am. J. Biochem. Biotechnol.* 4 101–104. 10.3844/ajbbsp.2008.101.104

[B12] Kim J. Y. Son M. J. Son C. Y. Radua J. Eisenhut M. Gressier F. (2019). Environmental risk factors and biomarkers for autism spectrum disorder: an umbrella review of the evidence. *Lancet Psychiatry* 6 590–600. 10.1016/s2215-0366(19)30181-6 31230684

[B13] Lakshmi Priya M. D. Geetha A. (2011). Level of trace elements (copper, zinc, magnesium and selenium) and toxic elements (lead and mercury) in the hair and nail of children with autism. *Biol. Trace Elem. Res.* 142 148–158. 10.1007/s12011-010-8766-2 20625937

[B14] Lord C. Elsabbagh M. Baird G. Veenstra-Vanderweele J. (2018). Autism spectrum disorder. *Lancet* 392 508–520. 30078460 10.1016/S0140-6736(18)31129-2 PMC7398158

[B15] Lutsenko S. Bhattacharjee A. Hubbard A. L. (2010). Copper handling machinery of the brain. *Metallomics* 2 596–608. 10.1039/c0mt00006j 21072351

[B16] Maenner M. J. Shaw K. A. Baio J. Washington A. Patrick M. DiRienzo M. (2020). Prevalence of autism spectrum disorder among children aged 8 years - autism and developmental disabilities monitoring network, 11 sites, United States, 2016. *MMWR Surveill. Summ.* 69 1–12. 10.15585/mmwr.ss6802a1 32214087 PMC7119644

[B17] Meguid N. A. Bjorklund G. Gebril O. H. Dosa M. D. Anwar M. Elsaeid A. (2019). The role of zinc supplementation on the metallothionein system in children with autism spectrum disorder. *Acta Neurologica Belgica* 119 577–583. 10.1007/s13760-019-01181-9 31302864

[B18] Mehdi Y. Hornick J. L. Istasse L. Dufrasne I. (2013). Selenium in the environment, metabolism and involvement in body functions. *Molecules* 18 3292–3311. 10.3390/molecules18033292 23486107 PMC6270138

[B19] Ming X. Johnson W. G. Stenroos E. S. Mars A. Lambert G. H. Buyske S. (2010). Genetic variant of glutathione peroxidase 1 in autism. *Brain Dev.* 32 105–109. 10.1016/j.braindev.2008.12.017 19195803

[B20] Ming X. Stein T. P. Brimacombe M. Johnson W. G. Lambert G. H. Wagner G. C. (2005). Increased excretion of a lipid peroxidation biomarker in autism. *Prostaglandins Leukot. Essent. Fatty Acids* 73 379–384. 10.1016/j.plefa.2005.06.002 16081262

[B21] Nam S. M. Kim J. W. Kwon H. J. Yoo D. Y. Jung H. Y. Kim D. W. (2017). Differential effects of low- and high-dose Zinc supplementation on synaptic plasticity and neurogenesis in the hippocampus of control and high-fat diet-fed mice. *Neurochem. Res.* 42 3149–3159. 10.1007/s11064-017-2353-2 28770438

[B22] Newschaffer C. J. Croen L. A. Daniels J. Giarelli E. Grether J. K. Levy S. E. (2007). The epidemiology of autism spectrum disorders. *Annu. Rev. Public Health* 28 235–258. 17367287 10.1146/annurev.publhealth.28.021406.144007

[B23] No Author List (2020). Corrigendum to differences in food consumption, and nutritional intake between children with autism spectrum disorders, and typically developing children: a meta-analysis. *Autism* 24 531–536. 10.1177/1362361319898028 31931626

[B24] Parikshak N. N. Luo R. Zhang A. Won H. Lowe J. K. Chandran V. (2013). Integrative functional genomic analyses implicate specific molecular pathways and circuits in autism. *Cell* 155 1008–1021. 10.1016/j.cell.2013.10.031 24267887 PMC3934107

[B25] Pozebon D. Scheffler G. L. Dressler V. L. (2017). Elemental hair analysis: a review of procedures and applications. *Anal. Chim. Acta* 992 1–23. 10.1016/j.aca.2017.09.017 29054142

[B26] R Core Team (2017). *R: A Language and Environment for Statistical Computing.* Vienna: R Foundation for Statistical Computing.

[B27] Rayman M. P. (2012). Selenium and human health. *Lancet* 379 1256–1268. 22381456 10.1016/S0140-6736(11)61452-9

[B28] Raymond L. J. Deth R. C. Ralston N. V. (2014). Potential role of selenoenzymes and antioxidant metabolism in relation to autism etiology and pathology. *Autism Res. Treat.* 2014:164938. 10.1155/2014/164938 PMC3966422 24734177

[B29] Rosenfeld C. S. (2015). Microbiome disturbances and autism spectrum disorders. *Drug Metab. Dispos.* 43 1557–1571. 10.1124/dmd.115.063826 25852213

[B30] Russo A. J. Devito R. (2011). Analysis of copper and zinc plasma concentration and the efficacy of zinc therapy in individuals with asperger’s syndrome, pervasive developmental disorder not otherwise specified (PDD-NOS) and Autism. *Biomark. Insights* 6 127–133. 22174567 10.4137/BMI.S7286 PMC3235993

[B31] Scheiber I. F. Mercer J. F. Dringen R. (2014). Metabolism and functions of copper in brain. *Prog. Neurobiol.* 116 33–57. 10.1016/j.pneurobio.2014.01.002 24440710

[B32] Skalny A. V. Simashkova N. V. Klyushnik T. P. Grabeklis A. R. Radysh I. V. Skalnaya M. G. (2017a). Assessment of serum trace elements and electrolytes in children with childhood and atypical autism. *J. Trace Elem. Med. Biol.* 43 9–14. 10.1016/j.jtemb.2016.09.009 27707611

[B33] Skalny A. V. Simashkova N. V. Skalnaya A. A. Klyushnik T. P. Bjorklund G. Skalnaya M. G. (2017b). Assessment of gender and age effects on serum and hair trace element levels in children with autism spectrum disorder. *Metab. Brain Dis.* 32 1675–1684. 10.1007/s11011-017-0056-7 28664504

[B34] Sogut S. Zoroglu S. S. Ozyurt H. Yilmaz H. R. Ozugurlu F. Sivasli E. (2003). Changes in nitric oxide levels and antioxidant enzyme activities may have a role in the pathophysiological mechanisms involved in autism. *Clin. Chim. Acta* 331 111–117. 10.1016/s0009-8981(03)00119-0 12691871

[B35] Solovyev N. D. (2015). Importance of selenium and selenoprotein for brain function: from antioxidant protection to neuronal signalling. *J. Inorg. Biochem.* 153 1–12. 10.1016/j.jinorgbio.2015.09.003 26398431

[B36] Steinbrenner H. Sies H. (2013). Selenium homeostasis and antioxidant selenoproteins in brain: implications for disorders in the central nervous system. *Arch. Biochem. Biophys.* 536 152–157. 10.1016/j.abb.2013.02.021 23500141

[B37] The Lancet, and Neurology. (2017). Investing in autism: better evidence for better care. *Lancet Neurol.* 16:251. 10.1016/s1474-4422(17)30049-2 28327327

[B38] Tick B. Bolton P. Happe F. Rutter M. Rijsdijk F. (2016). Heritability of autism spectrum disorders: a meta-analysis of twin studies. *J. Child Psychol. Psychiatry* 57 585–595. 10.1111/jcpp.12499 26709141 PMC4996332

[B39] Varcin K. J. Alvares G. A. Uljarevic M. Whitehouse A. J. O. (2017). Prenatal maternal stress events and phenotypic outcomes in autism spectrum disorder. *Autism Res.* 10 1866–1877. 10.1002/aur.1830 28681538

[B40] Vorstman J. A. S. Parr J. R. Moreno-De-Luca D. Anney R. J. L. Nurnberger J. I. Hallmayer J. F. (2017). Autism genetics: opportunities and challenges for clinical translation. *Nat. Rev. Genet.* 18 362–376. 10.1038/nrg.2017.4 28260791

[B41] Willsey A. J. Sanders S. J. Li M. Dong S. Tebbenkamp A. T. Muhle R. A. (2013). Coexpression networks implicate human midfetal deep cortical projection neurons in the pathogenesis of autism. *Cell* 155 997–1007. 10.1016/j.cell.2013.10.020 24267886 PMC3995413

[B42] Yui K. Kawasaki Y. Yamada H. Ogawa S. (2016). Oxidative stress and nitric oxide in autism spectrum disorder and other neuropsychiatric disorders. *CNS Neurol. Disord. Drug Targets* 15 587–596. 10.2174/1871527315666160413121751 27071787

