# Corrigendum: Evaluation of Zn, Cu, and Se Levels in the North American Autism Spectrum Disorder Population

**DOI:** 10.3389/fnmol.2022.831799

**Published:** 2022-02-14

**Authors:** Sunil Q. Mehta, Supriya Behl, Patrick L. Day, Adriana M. Delgado, Nicholas B. Larson, Lindsay R. Stromback, Andrea R. Huebner, Timothy R. DeGrado, Jessica M. Davis, Paul J. Jannetto, Flora Howie, Mukesh K. Pandey

**Affiliations:** ^1^Division of Developmental and Behavioral Pediatrics, Department of Pediatric and Adolescent Medicine, Mayo Clinic, Rochester, MN, United States; ^2^Department of Psychiatry and Psychology, Mayo Clinic, Rochester, MN, United States; ^3^Children's Research Center, Department of Pediatric and Adolescent Medicine, Mayo Clinic, Rochester, MN, United States; ^4^Metals Laboratory, Department of Laboratory Medicine and Pathology, Mayo Clinic, Rochester, MN, United States; ^5^Department of Quantitative Health Sciences, Mayo Clinic, Rochester, MN, United States; ^6^Divisions of Nuclear Medicine and Research, Department of Radiology, Mayo Clinic, Rochester, MN, United States; ^7^Division of Community Pediatric and Adolescent Medicine, Department of Pediatric and Adolescent Medicine, Mayo Clinic, Rochester, MN, United States

**Keywords:** autism spectrum disorder, Zn, Cu, Se, biometals

In the original article, there was a mistake in the footnotes for **Table 3** and **4** as published. Previously, all units were reported as (mcg/mL). However, serum selenium values should be reported in (ng/mL), and hair/nail samples should be reported in (mcg/g). The corrected footnotes appear below.

“**Table 3**. Median Cu, Zn, and Se levels for cases and controls, stratified by sex. Serum zinc and copper levels are presented in units mcg/mL. Serum selenium levels are presented in units ng/mL. Bolded *p*-value denotes significance.”

“**Table 4**. Median Se levels between cases and controls, stratified by sex. Hair and nail levels are presented in units mcg/g. Bolded *p*-value denotes significance. 66 participants provided hair samples (26 ASD, 40 controls), and 62 provided nail samples (23 ASD, 39 controls). Only one ASD female provided hair/nail samples in this study, therefore female-specific comparisons were not conducted.”

Additionally, in the original article, there was an error in the units of measurement that we reported in our results. Previously, all units were reported as (mcg/mL). However, serum selenium values should be reported in (ng/mL), and hair/nail samples should be reported in (mcg/g).

A correction has been made to the *Abstract*. The corrected paragraph is shown below.

“Metal ion dyshomeostasis and disparate levels of biometals like zinc (Zn), copper (Cu), and selenium (Se) have been implicated as a potential causative factor for Autism Spectrum Disorder (ASD). In this study, we have enrolled 129 children (aged 2–4 years) in North America, of which 64 children had a diagnosis of ASD and 65 were controls. Hair, nail, and blood samples were collected and quantitatively analyzed for Zn, Cu and Se using inductively coupled plasma mass spectrometry (ICP-MS). Of the analyzed biometals, serum Se (116.83 ± 14.84 ng/mL) was found to be significantly lower in male ASD cases compared to male healthy controls (128.21 ± 9.11 ng/mL; *p* < 0.005). A similar trend was found for nail Se levels in ASD (1.01 ± 0.15 mcg/g) versus that of controls (1.11 ± 0.17 mcg/g) with a *p*-value of 0.0132 using a stratified Wilcoxon rank sum testing. The level of Se in ASD cohort was co-analyzed for psychometric correlation and found a negative correlation between total ADOS score and serum Se levels. However, we did not observe any significant difference in Zn, Cu, and Zn/Cu ratio in ASD cases versus controls in this cohort of North American children. Further studies are recommended to better understand the biology of the relationship between Se and ASD status.”

A correction has also been made to *Results, Serum Analysis of Zn, Cu, and Se, Paragraphs 1 & 2*. The corrected paragraphs are shown below.

“Of the 129 children enrolled, 74 provided a blood sample (52 children with ASD and 22 controls). Overall and sex-stratified comparisons were made between cases and controls (Table 3). Of these comparisons, median serum Se levels were lower in cases than controls, with significant difference among males (*p* = 0.004). A Receiver Operating Characteristic (ROC) curve analysis Under Curve (AUC) of 0.760 (95% CI = [0.619–0.902]; Figure 1). Among controls, there was a significant difference in serum Se levels by sex (Table 3; 109.41 ng/mL in females vs. 128.21 ng/mL in males; *p* < 0.001).

There were no significant differences in median serum Zn or Cu levels. The Zn/Cu ratio was similarly evaluated with no significant differences between cases and controls.”

The authors apologize for this error and state that this does not change the scientific conclusions of the article in any way. The original article has been updated.

## Publisher's Note

All claims expressed in this article are solely those of the authors and do not necessarily represent those of their affiliated organizations, or those of the publisher, the editors and the reviewers. Any product that may be evaluated in this article, or claim that may be made by its manufacturer, is not guaranteed or endorsed by the publisher.

